# Editorial: The role of inflammation in organ injury

**DOI:** 10.3389/fimmu.2025.1717035

**Published:** 2025-10-22

**Authors:** Zhen Guo, Di Fan

**Affiliations:** ^1^ Department of Cardiology, Zhongnan Hospital of Wuhan University, Wuhan, China; ^2^ Institute of Myocardial Injury and Repair, Wuhan University, Wuhan, China

**Keywords:** inflammation, organ injury, sepsis, biomarkers, cytokines

Inflammation represents one of the most fundamental and evolutionarily conserved biological responses for host defense against pathogens and tissue injury (1). However, Inflammation can transition from a protective mechanism to a pathogenic one, contributing significantly to organ dysfunction and failure across diverse clinical contexts. This Research Topic was conceived to address the critical knowledge gaps in our understanding of how inflammatory processes contribute to organ dysfunction, with the dual aims of identifying novel therapeutic targets and advancing precision medicine approaches to inflammatory diseases.

The concept of systemic inflammatory response syndrome and its progression to a mechanism of multi-organ dysfunction has been extensively studied, yet the precise mechanisms by which local inflammatory responses propagate to cause distant organ failure remain incompletely understood. This Research Topic provides evidence for the interconnected nature of inflammatory responses across organ systems. Chang et al. show how co-infections in COVID-19 patients create a storm of inflammatory mediators, leading to coagulopathy and myocardial injury that significantly worsens patient outcomes. Their findings align with established concepts of cytokine storm syndrome but provide novel insights into how secondary infections can amplify the initial inflammatory insult (2). Similarly, Li et al. reveal the intricate relationship between metabolic dysfunction and inflammatory susceptibility in their study of obstructive jaundice, showing that cholestasis primes the inflammatory system for exaggerated responses to endotoxins. This work extends previous observations about the gut-liver axis and demonstrates how organ-specific dysfunction can systemically alter inflammatory responsiveness (3).

The respiratory system emerges as both a primary target and a driver of systemic inflammation in several studies within this Research Topic. The comprehensive analysis of sepsis-associated acute respiratory distress syndrome presented here builds upon decades of research into acute lung injury, providing updated perspectives on pathomechanisms and therapeutic approaches. The persistent challenges in managing SA-ARDS underscore the need for paradigm shifts in our approach to pulmonary inflammation, particularly given the heterogeneity of ARDS phenotypes and the limited success of traditional anti-inflammatory strategies (4). Suchankova et al. contribute a particularly valuable perspective by identifying CD44 as a reliable biomarker for pulmonary fibrosis progression, addressing a critical clinical need for early detection of fibrotic transformation. Their work connects cellular activation patterns with clinical outcomes, demonstrating how inflammatory cell activation leads to the uncontrolled extracellular matrix deposition characteristic of progressive fibrosis. This finding is particularly relevant given the growing recognition that many respiratory infections, including SARS-CoV-2, can result in long-term pulmonary complications (5).

The study by Wawryk-Gawda et al. on e-cigarette vapor exposure provides timely insights into emerging public health concerns, revealing that alternative smoking products can trigger robust inflammatory responses that may be distinct from those induced by traditional cigarette smoking. Their observation that inflammatory effects are largely reversible upon cessation, with the notable exception of certain cytokines that persist longer in traditional cigarette users, has important implications for public health policy and cessation strategies. This work contributes to our understanding of how different inflammatory triggers can produce overlapping yet distinct pathological signatures.

Cardiovascular inflammation represents another critical domain where this Research Topic makes significant contributions. Cao et al. employ sophisticated Mendelian randomization approaches to establish causal relationships between specific immune cell populations and heart failure risk, moving beyond simple associations to demonstrate true causal relationships. Their identification of 40 immunophenotypes with significant causal relationships to heart failure provides a roadmap for future therapeutic targeting and represents a significant advance in our understanding of immune-mediated cardiac dysfunction (6). The intersection of cardiovascular and immune systems is further explored by Kristoffersson et al., who reveal unexpected interactions between the renin-angiotensin system and complement activation. Their demonstration that renin can directly cleave C3 to generate bioactive complement fragments challenges traditional views of these systems as independent and suggests novel therapeutic opportunities at their interface.

The neuroinflammatory components of organ injury are addressed through multiple perspectives in this Research Topic. Tuz et al. examined hypercholesterolemia’s impact on post-stroke inflammation demonstrates how metabolic comorbidities can profoundly alter the brain’s inflammatory response to injury, leading to larger infarcts and worse outcomes. This work extends previous observations about the role of cholesterol in neuroinflammation and provides mechanistic insights into why patients with metabolic syndrome often experience worse stroke outcomes (7). Wu et al. provide a comprehensive analysis of diabetic neuropathic pain that delivers crucial insights into how chronic inflammatory states can lead to persistent neurological dysfunction, emphasizing the complex interplay between metabolic dysfunction, inflammatory mediators, and ion channel regulation in pain pathogenesis.

Renal inflammation represents a particularly well-characterized example of how inflammatory processes can lead to progressive organ dysfunction. Zhu et al. present groundbreaking work on HMGB1 lactylation and its role in driving the formation neutrophil extracellular traps in acute kidney injury, providing a mechanistic link between metabolic dysfunction and inflammatory organ damage. This work is particularly significant as it identifies lactate not merely as a metabolic byproduct but as an active participant in inflammatory signaling, challenging traditional views of metabolic-inflammatory interactions (8). The comprehensive review by Zhang et al. on IL-6 in diabetic kidney disease synthesizes extensive literature to demonstrate how this pleiotropic cytokine contributes to renal dysfunction through multiple pathways, including direct effects on glomerular and tubular cells, modulation of fibrotic responses, and regulation of metabolic pathways.

Several studies in this Research Topic identify novel inflammatory mediators and pathways that represent promising therapeutic targets. de Farias et al. provide an analysis of IL-17A in multisystem inflammatory syndrome in children that delivers crucial insights into pediatric inflammatory responses following SARS-CoV-2 exposure, demonstrating that this cytokine may be not only as mechanistic player but also a valuable prognostic marker. The finding that higher IL-17A levels are associated with increased mortality risk has immediate clinical implications and aligns with growing evidence for the role of Th17 responses in severe inflammatory syndromes (9). Chen et al. present innovative work on exosome-mediated siRNA delivery for intestinal ischemia-reperfusion injury, representing a convergence of mechanistic understanding with cutting-edge therapeutic approaches. Their use of milk-derived exosomes to deliver CCL7-targeting siRNA demonstrates how natural biological systems can be harnessed for precision therapeutic interventions.

The integration of computational approaches with traditional biological research is exemplified in Zhang et al.’s study of autoimmune-related genes in intracranial aneurysms, where machine learning algorithms identified ADIPOQ and IL21R as key diagnostic markers. This work demonstrates how artificial intelligence can enhance our ability to identify meaningful patterns in complex biological datasets and translate these findings into clinically useful tools (10). The application of multiple machine learning approaches provides confidence in the identified biomarkers while highlighting the potential for precision medicine approaches in inflammatory vascular diseases.

This Research Topic also addresses specialized inflammatory contexts that require particular attention. de Farias et al.’s study on MIS-C highlights how inflammatory responses can manifest differently across age groups, emphasizing the importance of age-specific research in inflammatory diseases. Pediatric inflammatory syndromes often present unique challenges due to developmental differences in immune function and the distinct pathogen exposure history of children compared to adults. Zhou et al.’s work on fungal keratitis and PPAR signaling demonstrates how tissue-specific factors can modulate inflammatory responses, with PPARs serving as master regulators of both inflammatory and metabolic processes in ocular tissues.

The therapeutic implications emerging from this body of work are substantial and multifaceted. The identification of specific inflammatory mediators such as IL-17A, IL-6, and CCL7 provides clear targets for precision anti-inflammatory therapies, moving beyond broad immunosuppression toward targeted interventions. Li et al.’s demonstration of combination therapeutic approaches, such as the synergistic effects of exercise preconditioning and resveratrol, suggests that optimal therapeutic outcomes may require multi-modal interventions that address different aspects of the inflammatory cascade. Novel drug delivery systems, particularly the exosome-based approaches demonstrated in this Research Topic, offer the potential for improved therapeutic specificity while reducing systemic side effects.

The biomarker discoveries presented throughout this Research Topic could enable more precise diagnosis and monitoring of inflammatory organ injury. The identification of CD44 in pulmonary fibrosis and IL-17A in MIS-C provides clinicians with tools for earlier detection and better risk stratification. These advances are particularly important given the often subtle early stages of inflammatory organ dysfunction and the critical importance of early intervention in preventing irreversible damage.

Looking forward, this Research Topic identifies several critical areas for future investigation. The dynamic interplay between metabolism and inflammation requires further mechanistic study, particularly as we recognize that many traditional metabolic pathways have previously unappreciated roles in inflammatory signaling. The development of personalized approaches to inflammatory disease management will require a deeper understanding of individual variations in the inflammatory responses, genetic susceptibility factors, and environmental modifiers. The integration of artificial intelligence and machine learning approaches with traditional biological research methods holds particular promise for identifying novel therapeutic targets and predicting treatment responses.

The work compiled in this Research Topic demonstrates that inflammation is far more than a simple response to injury – it is a complex, highly regulated process that can be both protective and destructive depending on the context, timing, and magnitude of the response. Understanding these nuances will be essential for developing the next generation of anti-inflammatory therapies and improving outcomes for patients with inflammatory organ injury.

## References

[1] Bender EC Tareq HS Suggs LJ. Inflammation: a matter of immune cell life and death. NPJ BioMed Innov. (2025) 2:7. doi: 10.1038/s44385-025-00010-4 PMC13055066 42032111

[2] Fajgenbaum DC June CH. Cytokine storm. N Engl J Med. (2020) 383:2255–73. doi: 10.1056/NEJMra2026131, PMID: 33264547 PMC7727315

[3] Albillos A de Gottardi A Rescigno M. The gut-liver axis in liver disease: Pathophysiological basis for therapy. J Hepatol. (2020) 72:558–77. doi: 10.1016/j.jhep.2019.10.003, PMID: 31622696

[4] Xu H Sheng S Luo W Xu X Zhang Z. Acute respiratory distress syndrome heterogeneity and the septic ARDS subgroup. Front Immunol. (2023) 14:1277161. doi: 10.3389/fimmu.2023.1277161, PMID: 38035100 PMC10682474

[5] George PM Wells AU Jenkins RG. Pulmonary fibrosis and COVID-19: the potential role for antifibrotic therapy. Lancet Respir Med. (2020) 8:807–15. doi: 10.1016/S2213-2600(20)30225-3, PMID: 32422178 PMC7228727

[6] Li J Liu L Luo Q Zhou W Zhu Y Jiang W. Exploring the causal relationship between immune cell and all-cause heart failure: a Mendelian randomization study. Front Cardiovasc Med. (2024) 11:1363200. doi: 10.3389/fcvm.2024.1363200, PMID: 38938655 PMC11210391

[7] Hansen SB Wang H. The shared role of cholesterol in neuronal and peripheral inflammation. Pharmacol Ther. (2023) 249:108486. doi: 10.1016/j.pharmthera.2023.108486, PMID: 37390970

[8] Zhang D Tang Z Huang H Zhou G Cui C Weng Y. Metabolic regulation of gene expression by histone lactylation. Nature. (2019) 574:575–80. doi: 10.1038/s41586-019-1678-1, PMID: 31645732 PMC6818755

[9] Korn T Bettelli E Oukka M Kuchroo VK. IL-17 and Th17 cells. Annu Rev Immunol. (2009) 27:485–517. doi: 10.1146/annurev.immunol.021908.132710, PMID: 19132915

[10] Rajkomar A Dean J Kohane I. Machine learning in medicine. N Engl J Med. (2019) 380:1347–58. doi: 10.1056/NEJMra1814259, PMID: 30943338

